# Long-Term Phenological Shifts in Butterfly Species from Transylvania, Romania—A Case Study

**DOI:** 10.3390/insects16101071

**Published:** 2025-10-20

**Authors:** Cristina Costache, László Rakosy, Demetra Rakosy

**Affiliations:** 1 Doctoral School of Integrative Biology, Faculty of Biology and Geology, Babeș-Bolyai University, Str. Republicii 44, 400015 Cluj-Napoca, Romania; 2 Department of Taxonomy and Ecology, Babeș-Bolyai University, Str. Republicii 48, 400015 Cluj-Napoca, Romania; 3 Department of Forestry and Environmental Protection, Ştefan cel Mare University, Str. Universităţii 13, 720225 Suceava, Romania; 4 Thünen Institute for Biodiversity, Bundesallee 65, 38116 Braunschweig, Germany

**Keywords:** historical data, decline, Romania, climate change, phenological cycles

## Abstract

**Simple Summary:**

Climate change can significantly impact insects, such as butterflies, by shifting their geographic ranges, altering the number of generations they produce annually, or changing their flight periods. To date, most evidence of these effects comes from Western and Central Europe, where long-term monitoring programs have provided the necessary data to detect climate-driven trends. In contrast, Eastern Europe—often considered a biodiversity haven due to the relatively lower rate of land-use change—has lacked such data, making it difficult to assess climate change impacts in the region. In this study, we utilize an alternative data source: historical museum collections combined with contemporary records and long-term climate data. This approach allows us to investigate how the phenological cycles of 16 butterfly species with varying life history traits have responded to climate change. Our findings reveal that butterfly species emerging in spring now begin their flight periods approximately 15 days earlier, while those active in autumn have extended their flight periods by an average of 23 days. These shifts in flight periods may have significant ecological consequences, particularly through altered synchrony with host plants and other critical resources.

**Abstract:**

Insects can respond rapidly to climate change through population fluctuations, range shifts, altered voltinism, life cycle changes, flight period adjustments, behavioural shifts, and changes in habitat or food preference—often varying by region due to local environmental and anthropogenic factors. While the phenological cycles of diurnal lepidopterans have been extensively studied in countries with large monitoring networks, eastern and southeastern Europe remain under-researched. This study provides the first insights into phenological shifts in 16 butterfly species in Cluj-Napoca (Transylvania, Romania) between 1921 and 2023, using a unique dataset combining historical and recent records. The species studied include spring-emerging, multivoltine, and migratory butterflies. Phenological trends were analyzed in relation to long-term climatic data. Results show that spring species now emerge approximately 15 days earlier, and autumn species extend their flight periods by up to 23 days. These changes correlate with multi-decadal trends in temperature and precipitation. We also discuss changes in voltinism and migratory behaviour and the potential impacts of climate change on butterfly populations in the study region.

## 1. Introduction

An increasing number of scientific papers are being published from all corners of the world, addressing the subject of climate change and its repercussions for insect populations [1,2,3,4,5,6,7]. Insects, such as Lepidoptera, exhibit high sensitivity to climate change, although the degree and direction of their responses may differ [8,9,10,11]. Some of the most discussed topics today are the impact of the annual increase in temperatures, earlier and persistent droughts, as well as warmer winters on lepidoptera populations [12,13,14,15,16,17]. Negative impacts, such as the overall decline of biomass of lepidopteran populations or even the extinction of certain species, has been demonstrated and brought to the attention of the scientific community and the general public repeatedly over the last 25 years [12,13,14,18]. Yet, positive effects have also been observed [8,11]. With changes in climate predicted to exacerbate over the next decades [19], there is a growing urgency to better understand the impact of climate change on Lepidoptera, and other insect groups. However, most studies to date are centred around similar geographic locations and habitats, mostly Western Europe [20,21,22,23,24,25,26] (but see [27,28,29,30]). More research is needed in understudied regions to highlight the impact of climate change on lepidopteran populations.

Phenological shifts in organisms serve as valuable bioindicators of climate change [31,32]. This is also true for Lepidoptera, which serve as valuable indicators for studying climate change due to their sensitivity to climatic and environmental variables, rapid responses, and extensive history of research [23,26,33,34]. As poikilothermic organisms, which evolved close relationships with plants both as adults and as larvae, Lepidoptera are highly influenced by variations in temperature and precipitation [7,35]. In England, a 1–1.5 °C increase in spring and summer temperatures was correlated with phenological changes by 6–8 days between 1976 and 1998 [17]. In the Mediterranean Basin too, a mean temperature increase of 1–1.5 °C in specific spring or summer months between 1988 and 2002 led to advances in the first appearance dates of all assessed butterfly species [36]. In contrast, higher precipitation led to a delay in the life cycles of butterflies [36]. Similar patterns have been observed across Europe, and are not restricted to butterflies, but also occur in moths [21,37,38].

Despite these overall consistent patterns, not all species respond to temperature changes in the same way, with responses varying depending on their evolutionary history, life cycle traits (overwintering stages, voltinism, etc.), as well as the state of the habitats they depend on and the extend of the induced climatic variation [8]. For example, a study on Edith’s checkerspot butterfly (*Euphydryas editha*) and the winter moth (*Operophtera brumata*), showed that species which evolved a less precise phenological match with their food plants may be more strongly affected by climate change [39]. In turn, others have found that butterflies that overwinter in the pupal stage are most affected by temperature changes [40,41]. Moderate warming can also initially benefit certain species by speeding up their development and broadening their distribution [42]. Unusual fluctuations in temperature, such as unexpectedly high or low temperatures, can also impact flight durations and the number of generations, a phenomenon that has been reported in the last 20–25 years [8,36].

In recent years, researchers from Western Europe have published their observations regarding correlations between phenological shifts in the life cycles of butterflies and climate change [9,23,43]. They have observed an extension of the period of flight, with earlier occurrences at the beginning of the season and later occurrences in the autumn. An earlier flight onset can lead to phenological mismatches between butterflies and their food plants, which may not have synchronized their phenological cycles accordingly, thereby affecting the survival of butterfly populations [43,44]. However, an extended flight period can allow many species to increase the number of generations produced per year [43]. Climate-induced shifts in phenological pattern can have seemingly beneficial effects, with enhanced late-season generations contributing to increases in the growth rates of overwintering populations [43]. However, these late generations may not always complete their development before winter, leading to “lost generations” [43]. Butterfly species have also been shown to either shift or expand their ranges northwards or to higher altitudes leading to changes in their distribution area, but potentially also a decline in species diversity at more southern locations or lower elevations [15,26,45,46]. These shifts can disrupt existing ecological relationships, as butterflies may not find suitable host plants or microhabitats in their new areas. Despite some positive effects of climate change, the combined pressures of climate change and habitat alteration are leading to significant decrease in butterfly population size [10,46,47,48].

However, the papers that have appeared on this topic are concentrated in countries that have a large amount of data, often obtained through citizen science [32]. But what is the situation in most other countries, where the number of volunteers monitoring butterflies and moths is low or very low? It is often suggested that the situation is better in eastern and southeastern European countries. Pollution and habitat destruction are more moderate, and insect declines are much lower than in central and western European countries. In reality, however, the situation in countries, such as Romania is no better than in Central European countries [34]. Due to the lack of extensive data provided by volunteers through citizen science, the phenological response of insects to climate change in countries that do not benefit from a large monitoring network is not known. However, historical data has the potential to fill in this knowledge gap [49,50]. In the present study we integrated all existing data—from historical collections to present day observations—on butterflies from the most studied area in Romania, located in Transylvania, around the city of Cluj-Napoca. Our aim was to analyze whether butterflies have undergone changes in their phenological cycles, whether these changes differ among species and finally, whether any shifts in the phenological cycles could be explained by climate change—defined as shifts in temperature averages, snow cover on the ground, and precipitation.

## 2. Materials and Methods

### 2.1. Study Area and Period

Long-term data on butterfly populations is rare in Romania, mainly due to the lack of national or regional butterfly monitoring programs. However, some areas of the country have benefited from decades of butterfly sampling, leading to considerable historic collections, not yet assessed for their value in examining long-term population trends in butterfly species. In the present study we focused on one such area surrounding the city of Cluj-Napoca (Transylvania).

Transylvania is Romania’s largest province, located at the centre of the country. It includes parts of the Carpathians and surrounding lowlands. Its varied topography and land-use create a mosaic of habitats, that support high butterfly diversity [51]. Long-standing low-intensity land-use has helped preserve species-rich habitats, though recent agricultural intensification and climate change increasingly threaten local butterfly populations. Cluj is one of the major cities of Transylvania, located in its north-western region. The climate of Cluj and its surroundings is temperate continental, with altitudes ranging between 350 and 800 m. The natural meso- and mesoxerophilous grasslands are used as pastures for sheep and cattle or as agricultural land. The few remaining deciduous forests are dominated by beech, hornbeam and oak.

The region of Cluj has a long history of lepidopterological studies (and the availability of long-term climate data), which allowed us to select three time periods for assessing the long-term impact of shifts in temperature and precipitation on the phenological cycles of butterflies. In the period before the Second World War and a few years after (1920–1948), several lepidopterologists were active in Cluj-Napoca (Transylvania), among which A. Ostrogovich [52] and St. Peterfi [53] were the most famous. These collectors have contributed to rich lepidopterological collections well preserved in the natural history museums in Cluj-Napoca (The Zoology Mueseum of the Babeş-Bolyai University) and Bucharest (The Grigore Antipa Natural History Museum). From these collections 202 specimens were included in the study, specimens which represented the first or last observed individual of the selected species for a particular year and not the entire collection. For the period 1965–1979 we used data from private collections and field observations of other lepidopterologists from Cluj, namely Carol Bere, Antal Kovrig, Rudolf Nemes and Vladimir Marg Manoliu (73 specimens). Finally, for the period 1980–2023 we used transect-based field observations conducted by Vladimir Marg Manoliu, Marin Goia, and László Rákosy (250 observations). The transects cover the same general area, as the historical collections. The data obtained from historical and present-day collections and observations, was complemented with phenological data for butterflies in the Cluj area extracted from the literature [53,54]. Although our initial aim was to analyze a century of data, the inability to verify the accuracy of earlier collection records and on limitations in the climate records led us to split the study period into two main time intervals 1921–1960 and 1973–2023.

### 2.2. Data Acquisition

#### 2.2.1. Butterfly Data

Historical collections and contemporary observations were used to determine the first annual recording dates of spring-emerging butterfly species, as well as the last annual recording dates for multivoltine species with extended flight periods, as documented in the Cluj-Napoca area. From the total of 95 butterfly species known to occur in the area [51,54] we selected 16 species, based on data availability, the period of emergence, the number of generations per year, length of the flight period and migratory behaviour. For species which emerge from the pupa in spring, 12 species were selected: *Erynnis tages* (Linnaeus, 1758), *Pyrgus malvae* (Linnaeus, 1758), *Iphiclides podalirius* (Linnaeus, 1758), *Papilio machaon* (Linnaeus, 1758), *Leptidea sinapis/juvernica* (Linnaeus, 1758), *Anthocaris cardamines* (Linnaeus, 1758), *Pieris napi* (Linnaeus, 1758), *Pieris rapae* (Linnaeus, 1758), *Colias alfacariensis/hyale* (Ribbe, 1905), *Glaucopsyche alexis* (Poda, 1761), *Plebejus argus* (Linnaeus, 1758), *Erebia medusa* ([Denis & Schiffermüller], 1775). The five butterfly species with multiple generations per year and a long flight period which extends towards autumn were: *Pieris rapae* (Linnaeus, 1758), *Colias alfacariensis/hyale* (Ribbe, 1905), *Colias croceus* (Geoffroy in Fourcroy, 1785), *Polyommatus icarus* (Rottemburg, 1775) and *Coenonympha pamphilus* (Linnaeus, 1758). Finally, a migratory species, *Vanessa atalanta* (Linnaeus, 1758) was chosen, which previously was not known to overwinter as an adult in Romania, but which is expected to do so under changing climatic conditions change. Each observation was annotated with species name (genus, species) and date of observation (day, month, year). To standardize the data, only the first or the last date the species was observed was selected, with a single record. An overview of the sample size and the species considered is given in Table 1.

#### 2.2.2. Climate Data

For both study periods, we used monthly average data for temperature (°C) and total monthly precipitation (L/m^2^), as these two climatic parameters are essential for influencing the emergence dates of butterflies [55], their flight duration as well as overwintering patterns [36,40]. For a more precise interpretation of the observed phenological shifts in butterfly species, we also used the snow cover data, as snow cover impacts both the overwintering larvae and adults [56]. Precipitation and temperature data were collected for the period 1921 to 2023. While average annual data was available for the whole period, the average monthly precipitation and temperature could only be assessed for the period of 1973–2023. Snow cover data were also available only from 1973 to 2023. For the first period, spanning from 1921 to 1960, we extracted data from climate catalogues, while for the period from 1973 to 2023, data were extracted from the private archives provided by request by the National Meteorological Agency in Romania. The data on climate, spanning the period from 1973 to 2023, were collated from a single meteorological station situated in Cluj County. The geographical location of this station is specified by the coordinates 46°46′39.974″ N, 23°34′16.699″ E, with an altitude of 410 m, and it is located 1.5 km northwest of the city of Cluj-Napoca.

### 2.3. Statistical Analyses

We used Python (version 3.13) to process and visualize the data with the pandas, matplotlib, and seaborn libraries. We generated Kernel density estimation (KDE) plots to visualize changes in phenological patterns between 1921 and 2023 by overlaying species-specific scatter points, colour-coded by genus. To illustrate the changes in phenological cycles of butterfly species we calculated deviations from the long-term species-specific average appearance date, visualized as bar plots using matplotlib.pyplot.bar(), with earlier and later appearances colour-coded accordingly. We chose to aggregate the date on decades for datapoint between 1973 and 2023 only to reduce interannual variability and emphasize long-term climatic and phenological trends. The reduced time span, relative to the Kernel Density Estimation (KDE) plots, resulted from an inadequate sample size for the 1921–1973 period. We opted for a visual assessment of patterns, rather than formal significance testing due to the limitations in the quantity and distribution of the data.

For the period 1921–1973 annual average temperature and precipitation was calculated based on the available data. For the period 1973–2023, average monthly temperature and precipitations were analyzed using data from the Cluj-Napoca meteorological station, merged by decade as a heatmap, using seaborn.heatmap() to highlight seasonal and long-term warming trends. We combined data on decades to show long-term trends while minimizing the short-term fluctuations in weather, local microclimate effects, and observational inconsistencies for a clear visualization of climate change rather than episodic weather events. We have also analyzed the trend and the impact of temperature on the snow cover duration. In order to observe this relationship, a linear regression was performed with scipy.stats.linregress(). The resulting scatterplot was visualized using matplotlib, with points colour-coded for each decade and the coefficient of determination (R^2^).

## 3. Results

### 3.1. Species with Emergence in Spring

**Figure 1:**
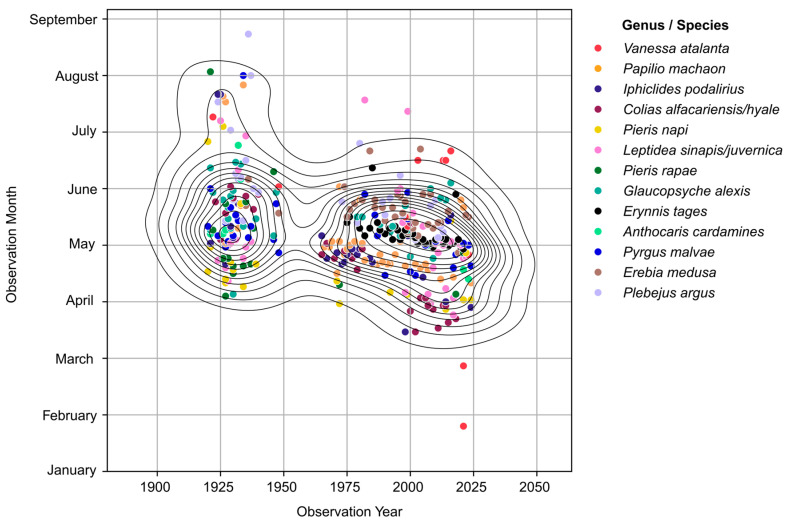
Phenological trends in the butterfly species which emerge in spring from 1921 to 2023. Individual species are represented by different colours in the scatterplot and are part of the cumulative data analyses for phenological shifts. Lines represent the KDE (kernel density estimation) fit through the data, representing a 2D distribution of the data.

**Figure 2:**
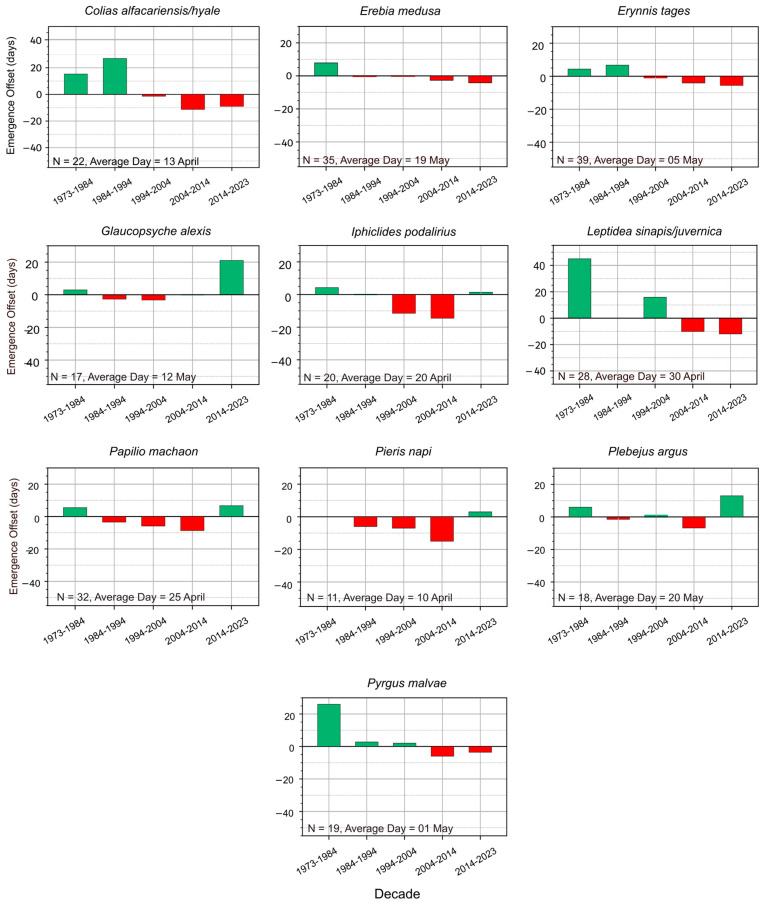
Deviation of first observation dates of the 11 spring emerging species from the long-term mean of the first observation (N = sample size; Average day = average day of first emergence). Green bars represent later sightings = positive deviation; red bars represent earlier sightings = negative deviation. The time interval between 1973 and 2023 was split by decades in order to highlight breaking points in the observed trends.

From the eleven butterfly species which emerge in spring, five species exhibited phenological shifts in more recent records (post-1973), as evidenced by a concentrated cluster of observations occurring earlier in the season (Figure 1). The observations of the species *P. napi*, *C. alfacariensis/hyale* and *L. sinapis/juvernica*, show a drastic shift, with peak observations moving from May to April and mid-March over the same time frame (Figure 2). Data after 1973 being more numerous, the trend change is more evident for the last 40 years. Not all species, however, show clear phenological shifts between 1973 and 2023, with some species exhibiting smaller or less consistent changes. For example: *P. malvae* and *E. medusa* show only moderate shifts in their phenological cycles (Figure 2). *G. alexis* and *E. tages* appear to show relatively stable emergence times, with peak first observations remaining from late April to mid-May (Figure 2).

### 3.2. Species with Multiple Generations and Long Flight Periods

**Figure 3:**
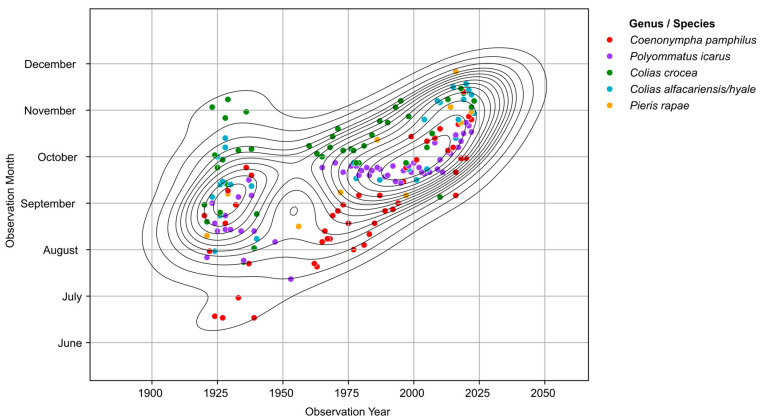
Phenological trends in butterfly species with multiple generations and long flight periods from 1921 to 2023. The individual species are represented by different colours in the scatterplot and are part of the cumulative data analyzed for phenological shifts. Lines represent the KDE (kernel density estimation) fit through the data, representing a 2D distribution of the data.

**Figure 4:**
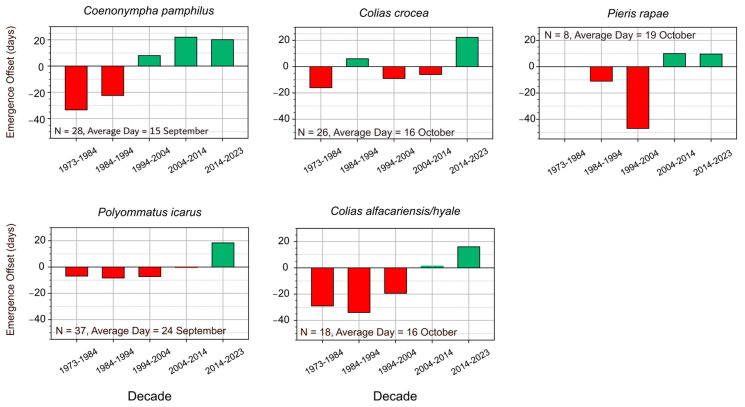
Deviation of last observation dates of 5 species with multiple generations and long flight periods from the long-term mean of the last observation (N = sample size; Average day = average day of last observation). Green bars represent later sightings = positive deviation; red bars represent earlier sightings = negative deviation. The time interval between 1973 and 2023 was split by decades in order to highlight breaking points in the observed trends.

The five species of butterflies chosen to capture the possible shift in the phenological cycles for multivoltine butterflies, showed a real prolongation of the flight period towards the end of October and beginning of November. The shift seems to have started around the 1970s–1980s and had a more pronounced shift in recent years (after the 2000s) (Figure 3). For *C. crocea*, *P. icarus* and *C. pamphilus*, the extending of the flight period towards the end of October is documented for the whole period 1973–2023 (Figure 4). *P. rapae* extends its flight period by more than 35 days between 1973 and 2023 (Figure 4). The most profound phenological shift is recorded in the *C. alfacariensis/hyale* complex, which extended its flight period from mid-September to early November between 1980 and 2023 (Figure 4).

### 3.3. Migratory Species

**Figure 5:**
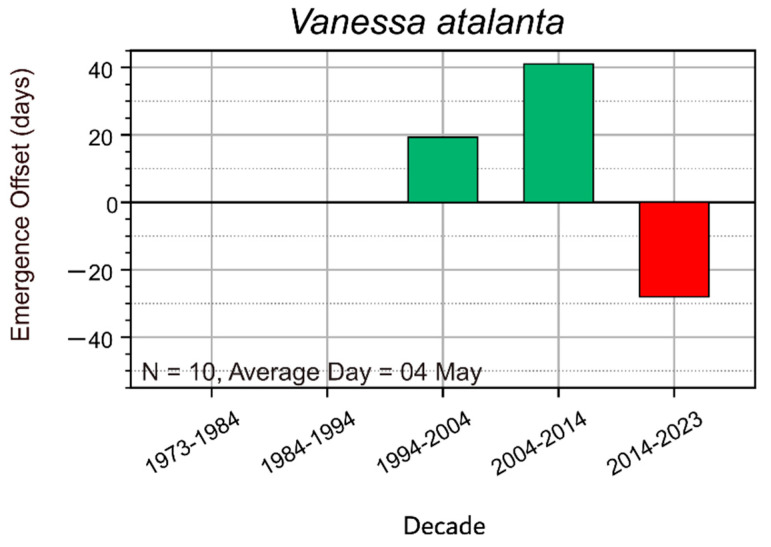
Deviation of last observation dates of the migratory species, *V. atalanta* from the long-term mean of the last observation (N = sample size; Average day = average day of last observation). Green bars represent later sightings = positive deviation; red bars represent earlier sightings = negative deviation. The time interval between 1973 and 2023 was split by decades in order to highlight breaking points in the observed trends.

*V. atalanta* was not previously known to overwinter north of the Southern Carpathians. Earlier records from Transylvania showed that the first individuals appeared in collections only after the 15th or 20th of May. Although it had been suspected that *V. atalanta* might be able to overwinter in the southern and southeastern parts of the country, no concrete evidence supported this assumption. However, over the past 15 years, sightings of *V. atalanta* in Cluj have become increasingly common in April and, in the last 5–6 years, even as early as March. This suggested the possibility that the species was beginning to overwinter in Transylvania, likely due to the region’s increasingly mild winters. These assumptions were confirmed when specimens were observed as late as mid-December during unusually warm days over the past 4–5 years (Figure 5).

### 3.4. Climate

**Figure 6:**
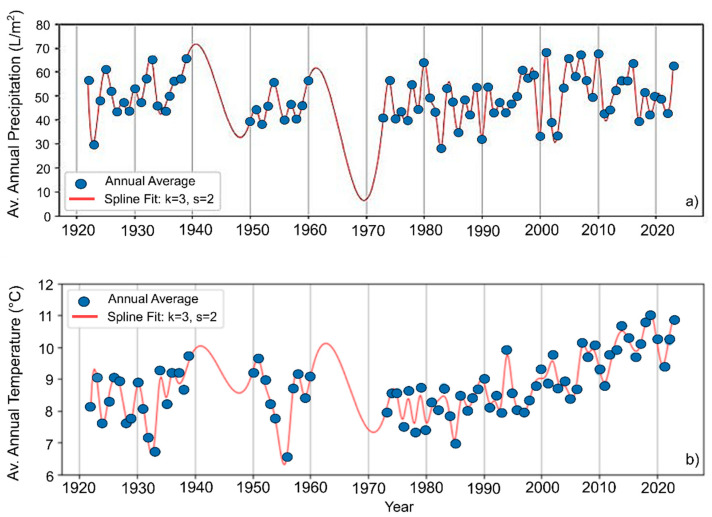
(**a**) Trend line of average precipitations (dark orange line) for the period 1921–2023. (**b**) Trend line of average temperatures (light orange line) for the period 1921–2023.

**Figure 7:**
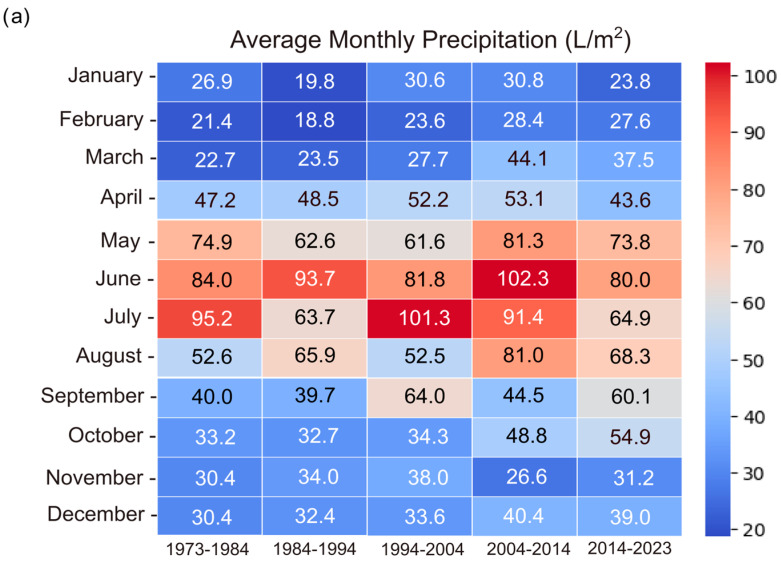

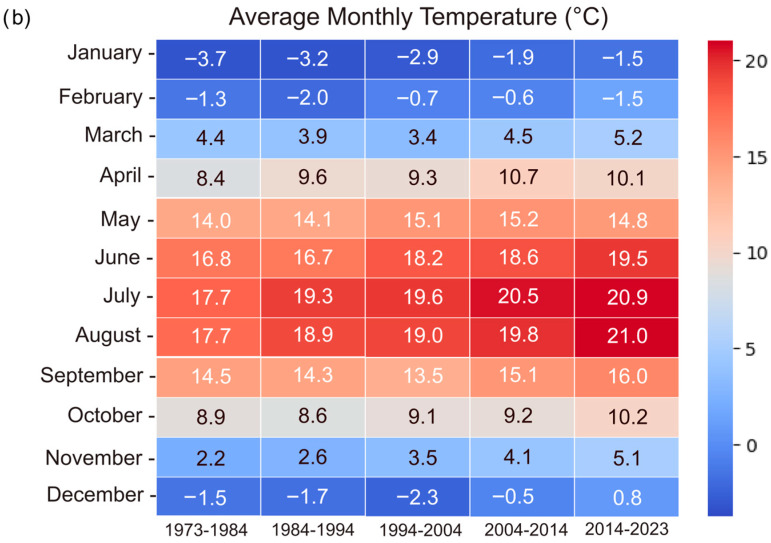
(**a**) Long-term temporal trends in average monthly precipitations (L/sq. m.) for the period 1973–2023 in the Cluj area. (**b**) Long-term trends in average monthly temperatures (°C) for the period 1973–2023 in the Cluj area.

In order to observe the phenological shifts in butterfly species, three major climatic parameters were extracted: the monthly average precipitations, the average temperature, and the snow cover for Cluj and its surroundings. The recorded annual precipitation fluctuated; however, the long-term mean annual precipitation remained relatively constant, with values between −11.20 and 21.40 (Figure 6a and Figure 7a). A clear and consistent warming trend was observed when analyzing the mean annual monthly temperatures, with the warming becoming more pronounced from approximately 1980 onward (Figure 6b). The warming of the climate becomes more evident if only the summer months are assessed, with average temperatures increasing significantly, peaking in July and August between 2014 and 2023 at 20.9 °C and 21.0 °C (Figure 7b).

In order to correlate the life cycle changes in butterfly species with the change in climatic conditions in winter, we also extracted the snow cover dynamics for the studied interval (Figure S1). This shows a clear negative linear trend, with snow cover days declining from approximately 66 days in 1973–1984 to around 36 days in 2014–2023, as average temperatures increased by roughly 2 °C. The regression line highlights this trend, with a high coefficient of determination (R^2^ = 0.97), indicating a robust correlation between rising temperatures and decreasing snow cover.

## 4. Discussions

In the absence of a standardized monitoring program for butterflies in Romania, we used all available historical and current data for one of the best studies areas of the country (Cluj-Napoca and its surroundings, Transylvania) from 1921 to 2023 to assess whether butterfly species have undergone changes in their phenological cycles due to climate change. The results show a phenological shift towards March-February for butterflies hatching from pupae in spring and a shift in the flight period towards October-November for species with multiple generations and long flight periods. This prolongation of the flight period towards the autumn was, most likely, associated an increase in voltinism, with some monovoltine species becoming bivoltine and some bivoltine species becoming consistently trivoltine. In the case of the migratory species *V. atalanta*, it has also been shown that it can successfully overwinter in Transylvania under the winter conditions of the last 10–15 years.

While there are relatively few studies conducted in Eastern Europe, Askeyev et al. [56] observed that there are definite differences in phenological patterns between Eastern and Western Europe, with Eastern regions exhibiting unique responses to climatic variations [57]. They noted that, even though many species are increasing their phenological occurrences, the degree and type of these changes can vary significantly based on local environmental conditions and species traits [56,57]. The variability in responses exhibited by different species of butterflies underscores the intricate nature of these interactions. Notably, certain species may either experience an early or delayed emergence or demonstrate no substantial alterations at all [41,58]. Phenological changes driven by climatic warming cannot be correlated with phylogenetic aspects in diurnal [41] or nocturnal Lepidoptera [38], the response being a species characteristic (species-related) and not a kinship (phylogenetic) one [59]. Previous studies have identified specific characteristics, such as the overwintering strategy or voltinism, that better predict how species will respond to climatic shifts [41,60,61,62].

Butterfly species which overwinter in the pupal stage, followed by those overwintering as larvae, have been thus shown to be more sensitive to climatic changes and to show more pronounced phenological shifts [17,63]. The response of species overwintering as eggs is slower to climatic changes, with phenological time lags being smaller, but species hibernating as adults have the fastest response, with their awakening to activity occurring as soon as the temperature becomes acceptable. Some authors consider the higher reaction speed of species overwintering as adults as an evolutionary advantage [64]. Rapid climatic warming associated with the awakening from hibernation of adults can only be considered an evolutionary advantage if synchronization with the trophic base (tree sap, nectar) is achieved. However, most of the time, the awakening of adults from hibernation during winter (December–February) does not coincide with the trophic supply, and therefore, the phenomenon is most likely disadvantageous [49,65,66]. In contrast, studies have shown that the extension of the flight period, can increase the number of generations multivolitine species can successfully produce, leading to population increases [43]. These positive effects of phenological shifts do not, however, seem to be able to counteract the negative impacts of climate change [43]. More research is thus needed to understand how and when particular butterfly species and communities respond to climate change.

In the present study an increase of +2 °C over the past 40 years was found, aligning with global observations of anthropogenic climate change during the latter half of the 20th century and early 21st century [36]. The increase in temperature was, however, not associated with any detectable changes in precipitation for the study region [67]. Instead, a strong decrease in the days with snow cover was found. The impact of these climatic changes was evident in the analyzed butterfly species, with the emergence and activity of first-generation butterflies becoming earlier from year to year, with the emergence dates shifting towards March-February by 2–3 weeks, compared to the 1921–1980 interval. For the species *P. machaon*, *I. podalirius* and the *C. alfacariensis/hyale* species complex, the phenological shift observed was over 20 days. The impact of earlier emergence dates on the populations of these species can manifest in multiple ways. The exposure of diapausing butterflies to repeated intervals of high temperatures during winter followed by late spring frosts have unfavourable effects on all overwintering stages, disrupting their normal life cycle, leading to earlier emergence of adults from overwintering pupae or premature activity in overwintering larvae and increased mortality rates when conditions reverted to colder temperatures [14,47]. The weakening of the organism following repeated metabolic activations during winter may also have implications on female (and male) reproductive selectivity (selection on mate discrimination) [12,68]. Such ecological changes caused by climate change have so far been little studied but could influence the decline of butterfly diversity.

Repeated warm periods during winters that have become much milder can also generate a ‘trap’ effect of climate warming, when due to higher temperatures, overwintering larvae become active but are out of synchronization with the trophic base [43,69,70,71]. The same can happen when larvae hatch too early or when adults start foraging before their feeding resource is available. In the much warmer winters of the last few years, the overwinter stages may awaken several times between December–February, which results in the consumption of energy reserves (especially lipids) without the possibility of replenishing them [43]. After several such awakenings followed by an unsuccessful attempt to find food sources, the organism loses the reserves needed for overwintering. In such situations, the larvae pupate undernourished or die before the trophic supply appears [32,70]. Lepidoptera hatched from such pupae are weakened, mate discrimination is impaired, and the females produce and lay fewer eggs. Similarly, in species that overwinter as adults, winter awakenings and unsuccessful feeding attempts reduce energy reserves [43,63]. As a result, butterflies emerge from the winter diapause, weakened, with low fertility, which will have repercussions on the following generations. If this phenomenon repeats itself from one year to the next, the impact on the populations is likely to be increasingly drastic, leading to the decline of the affected populations. Observational data on both diurnal and nocturnal species, support this. Thus, butterfly species such as *V. atalanta*, *Aglais urticae*, *Aglais io* or *Nymphalis polychloros* were occasionally observed on warm and sunny winter days, looking for, not yet available, food sources. Similarly, the moth genera *Xylena*, *Orthosia*, *Conistra*, *Litophane*, *Eupsilia*, *Jodia* (Fam. Noctuidae) were frequently observed in light traps placed in warm nights in December–February in the forests around Cluj-Napoca.

The consequences of climate change on the phenological cycles of butterflies can also extend to evolutionary dynamics and species distributions [57,72]. In the context of climate change, species may undergo rapid evolutionary changes, particularly those with shorter generation time [73]. This evolutionary pressure may lead to altered life history traits, such as changes in voltinism or reproductive strategies, which could help species cope with the challenges posed by climate change [14,74,75]. Nonetheless, the adaptability of species is not the same, and some may face greater risks of extinction if they cannot keep pace with the rapid environmental changes [60,73]. Changing overwintering conditions may also allow species which are adapted to milder conditions to persist in new areas. Our results indicate that species active in late summer can extend their flight period considerably into late autumn and early winter adapting to milder conditions in the winter or increase the number of generations produced per year, such as the *C. alfacariensis/hyale* complex, which extended its flight period by 45 days, from mid-September to early November between 1980 and 2023. This suggests that at least for some species, increasing temperatures may show positive effects.

An intriguing observation has been made for *V. atalanta*, a migratory species, which until 1990 failed to overwinter as an adult in Transylvania. Between 1921 and 1990, there are no records of hibernating or other, active, early spring individuals before May in Romania. After 1990, but especially after 2000, active specimens of *V. atalanta* were observed in Transylvania both on warm days in December and in February and March, which indicates the possibility of that the species may be overwintering under the warmer and shorter winters in Transylvania. From May onwards, overwintering specimens are joined by migrated specimens from the south. It is likely that the wintering possibilities for adult *V. atalanta* in the south of the country are much earlier, but we do not have such data or observations.

Changes in voltinism over the last 20 years have also been observed in some other butterfly species in Transylvania. Thus, *Apatura ilia* has changed its annual life cycle, becoming regionally bivoltine as in France, Germany, Austria and Hungary [76,77,78,79]. Until 2015, no specimens were observed that could be attributed to a second generation. After 2015, such records started to appear, and since 2023 the second generation is constant and stable. In Romania, south of the Carpathians as well as in Hungary, *A. ilia* has been bivoltine for many years. Other species, in which the 2nd or 3rd generation was only partial, have become constant and complete in the last 10 years. The increase in the number of generations can be seen as an adaptation to the prolonged availability of resources. So far there is no information to support the positive or negative effect of extending the flight period into the winter months or increasing the number of generations. Apparently, these changes can be considered as adaptive phenomena with positive effects. However, we cannot exclude the negative aspect, i.e., the trap effect, in which, due to certain unfavourable climatic conditions, additional generations have a low number of offspring [73]. The same can happen in the case of species that extend their period of activity into the winter months.

The findings showed in the present study fit well with similar research conducted across Europe. Scientists have found that in Germany, Austria, the Mediterranean region, and the UK, butterflies are affected by climate warming through a shift in the onset of their flight period and an increase in the number of generations per year [12,36,73,80,81,82]. However, while our study builds on an extensive dataset and highlights the impact climate change can have on butterfly species in Transylvania, it is limited by a rather narrow geographic and environmental range. Further studies are needed to expand the approach presented here to other areas in Romania and to broader environmental gradients (i.e., altitudinal or land-use gradients). For a better understanding of the response of lepidopterans to climate change (and other factors), a standardized monitoring network for both butterflies and moths is needed. So far, such a program is in its infancy in Romania, but may develop in the near future, led by the European Butterfly Monitoring Scheme [83]. To draw general conclusions about the response butterflies to climate change, modelling and predictions based on are needed solid regional knowledge, which can then be generalized to create scenarios for the future development of butterfly and moth populations and drive conservation action [57,61].

## 5. Conclusions

This study is among the few that, in the absence of long-term monitoring programs, combines historical and contemporary to assess how butterfly phenological patterns are shifting in response to climate change in Eastern Europe. We show that during the last decades climate change appears to have altered both the onset and end of butterfly flight periods and potentially to enable previously transitory species to establish viable populations. However, further research across broader environmental gradients is needed to fully capture the range of species-specific responses, both positive and negative, and to guide conservation prioritization effectively.

More broadly, our findings, along with those from other recent studies in Eastern Europe, challenge the long-standing perception that the region remains a safe haven for biodiversity due to historically lower rates of land-use change. While Eastern Europe may face somewhat different pressures than Western Europe, increasing evidence shows that both climate and land-use change are already impacting its once well-preserved biodiversity.

## Figures and Tables

**Table 1:** Overview of the assessed species and records per time period 1921–2023. Note that only those records were considered which reflected the first or last date the species was observed (a single record/species/year).

Species	Group	Total
*Anthocaris cardamines*	Spring emerging	14
*Colias alfacariensis/hyale*	Spring emerging	35
*Erebia medusa*	Spring emerging	41
*Erynnis tages*	Spring emerging	39
*Glaucopsyche alexis*	Spring emerging	40
*Iphiclides podalirius*	Spring emerging	34
*Leptidea sinapis*	Spring emerging	44
*Papilio machaon*	Spring emerging	46
*Pieris napi*	Spring emerging	28
*Pieris rapae*	Spring emerging	23
*Plebejus argus*	Spring emerging	31
*Pyrgus malvae*	Spring emerging	40
*Coenonympha pamphilus*	Multivolitin, long flight period	49
*Colias croceus*	Multivolitin, long flight period	50
*Colias alfacariensis/hyale*	Multivolitin, long flight period	29
*Pieris rapae*	Multivolitin, long flight period	16
*Polyommatus icaurs*	Multivolitin, long flight period	54
*Vanessa atalanta*	Migratory	13
Total		626

## Data Availability

The original contributions presented in this study are included in the article/Supplementary Materials. Further inquiries can be directed to the corresponding author.

## Supplementary Materials

The following supporting information can be downloaded at: https://www.mdpi.com/article/10.3390/insects16101071/s1, Figure S1: Relationship between the decline in snow cover and increasing average annual temperatures across decades (1973–2023). The interrupted line represents the linear fit through the data with a coefficient of determination R^2^ proving a strong negative correlation. Table S1: Raw data on spring emerging species and species with multiple generations and long flight periods, as well as climate data and essential metadata.

## References

[1] Vitali F., Habel J.C., Ulrich W., Schmitt T. (2023). Global Change Drives Phenological and Spatial Shifts in Central European Longhorn Beetles (Coleoptera, Cerambycidae) during the Past 150 Years. Oecologia.

[2] Forrest J.R. (2016). Complex Responses of Insect Phenology to Climate Change. Curr. Opin. Insect Sci..

[3] Bartomeus I., Ascher J.S., Wagner D., Danforth B.N., Colla S., Kornbluth S., Winfree R. (2011). Climate-Associated Phenological Advances in Bee Pollinators and Bee-Pollinated Plants. Proc. Natl. Acad. Sci. USA.

[4] Hassall C., Owen J., Gilbert F. (2017). Phenological Shifts in Hoverflies (Diptera: Syrphidae): Linking Measurement and Mechanism. Ecography.

[5] Johansson F., Orizaola G., Nilsson-Örtman V. (2020). Temperate Insects with Narrow Seasonal Activity Periods Can Be as Vulnerable to Climate Change as Tropical Insect Species. Sci. Rep..

[6] Müller J., Hothorn T., Yuan Y., Seibold S., Mitesser O., Rothacher J., Freund J., Wild C., Wolz M., Menzel A. (2024). Weather Explains the Decline and Rise of Insect Biomass over 34 Years. Nature.

[7] Rumohr Q., Baden C.U., Bergtold M., Marx M.T., Oellers J., Schade M., Toschki A., Maus C. (2023). Drivers and Pressures behind Insect Decline in Central and Western Europe Based on Long-Term Monitoring Data. PLoS ONE.

[8] Hufnagel L., Kocsis M. (2011). Impacts of Climate Change on Lepidoptera Species and Communities. Appl. Ecol. Environ. Res..

[9] Sánchez-Bayo F., Wyckhuys K.A.G. (2019). Worldwide Decline of the Entomofauna: A Review of Its Drivers. Biol. Conserv..

[10] Cayton H.L., Haddad N.M., Gross K., Diamond S.E., Ries L. (2015). Do Growing Degree Days Predict Phenology across Butterfly Species?. Ecology.

[11] Birch R., Nebel L., Chittaro Y., Hermann G., Trusch R., Gelbrecht J., Markl G. (2025). The Diverse Reactions of Butterflies and Zygaenids (Lepidoptera) to Climate Change—A Large Scale, Multi-Species Study. Glob. Ecol. Biogeogr..

[12] Barve N. (2012). Climate-Change and Mass Mortality Events in Overwintering Monarch Butterflies. Rev. Mex. Biodivers..

[13] Habel J.C., Trusch R., Schmitt T., Ochse M., Ulrich W. (2019). Long-Term Large-Scale Decline in Relative Abundances of Butterfly and Burnet Moth Species across South-Western Germany. Sci. Rep..

[14] Hallmann C.A., Sorg M., Jongejans E., Siepel H., Hofland N., Schwan H., Stenmans W., Müller A., Sumser H., Hörren T. (2017). More than 75 Percent Decline over 27 Years in Total Flying Insect Biomass in Protected Areas. PLoS ONE.

[15] Warren M.S., Maes D., Van Swaay C.A.M., Goffart P., Van Dyck H., Bourn N.A.D., Wynhoff I., Hoare D., Ellis S. (2021). The Decline of Butterflies in Europe: Problems, Significance, and Possible Solutions. Proc. Natl. Acad. Sci. USA.

[16] Buckley L.B. (2022). Temperature-Sensitive Development Shapes Insect Phenological Responses to Climate Change. Curr. Opin. Insect Sci..

[17] Costache C., Crişan A., Rákosy L. (2021). The Decline of Butterfly Populations Due to Climate and Land Use Change in Romania. Climate and Land Use Impacts on Natural and Artificial Systems.

[18] Habel J.C., Schmitt T., Gros P., Ulrich W. (2024). Active around the Year: Butterflies and Moths Adapt Their Life Cycles to a Warming World. Glob. Change Biol..

[19] Calvin K., Dasgupta D., Krinner G., Mukherji A., Thorne P.W., Trisos C., Romero J., Aldunce P., Barrett K., Blanco G., Lee H., Romero J., Core Writing Team (2023). IPCC, 2023: Climate Change 2023: Synthesis Report. Contribution of Working Groups I, II and III to the Sixth Assessment Report of the Intergovernmental Panel on Climate Change.

[20] Hickinbotham E.J., Pattison Z., Fox R., Rushton S.P. (2024). Drivers of Moth Phenology in England and Wales. J. Insect Conserv..

[21] Bonelli S., Cerrato C., Barbero F., Boiani M.V., Buffa G., Casacci L.P., Fracastoro L., Provenzale A., Rivella E., Zaccagno M. (2021). Changes in Alpine Butterfly Communities during the Last 40 Years. Insects.

[22] Hill G.M., Kawahara A.Y., Daniels J.C., Bateman C.C., Scheffers B.R. (2021). Climate Change Effects on Animal Ecology: Butterflies and Moths as a Case Study. Biol. Rev..

[23] McDermott Long O., Warren R., Price J., Brereton T.M., Botham M.S., Franco A.M.A. (2017). Sensitivity of UK Butterflies to Local Climatic Extremes: Which Life Stages Are Most at Risk?. J. Anim. Ecol..

[24] Miao B., Peng Y., Yang D., Kubota Y., Economo E.P., Liu C. (2021). Climate and Land-use Interactively Shape Butterfly Diversity in Tropical Rainforest and Savanna Ecosystems of Southwestern China. Insect Sci..

[25] Ren J., Li S., He M., Zhang Y. (2022). Butterfly Community Diversity in the Qinling Mountains. Diversity.

[26] Santorufo L., Ienco A., Scalercio S. (2021). Climate Warming Drives Divergence of Montane Butterfly Communities in Southern Italy. Reg. Environ. Change.

[27] Knyazev S.A., Saikina S.M., Teploukhov V.Y., Sitnikov P.S., Galich D.E., Kosterin O.E. (2024). First Records of *Apatura ilia* ([Denis & Schiffermüller], 1775) and *Limenitis camilla* (Linnaeus, 1764) in West Siberia. Acta Biol. Sib..

[28] Yakovlev R.V., Kostyunin A.E. (2015). Range Expansion of *Apatura iris* (Linnaeus, 1758) in Siberia (Lepidoptera: Nymphalidae). SHILAP Rev. Lepidopterol..

[29] Gordeev S.Y., Gordeeva T.V., Korsun O.V. (2023). On the Reasons of *Limenitis sydyi* (Lepidoptera, Nymphalidae) expansion in Transbaikalia. Russ. J. Biol. Invasions.

[30] Gordeev S.Y., Gordeeva T.V. (2020). The Causes of Penetration of *Apatura* Fabricius, 1807 (Lepidoptera, Nymphalidae) Species into Western Transbaikalia. Russ. J. Biol. Invasions.

[31] Shahin H., Correia A.H., Orazio C., Branco M., Almeida M.H. (2019). Monitoring Two REINFFORCE Network Arboreta: First Result on Site, Climate and Genetic Interaction Showing Impact on Phenology and Biotic Damages. Sci. For..

[32] Macgregor C.J., Thomas C.D., Roy D.B., Beaumont M.A., Bell J.R., Brereton T., Bridle J.R., Dytham C., Fox R., Gotthard K. (2019). Climate-Induced Phenology Shifts Linked to Range Expansions in Species with Multiple Reproductive Cycles per Year. Nat. Commun..

[33] Walther G.-R., Post E., Convey P., Menzel A., Parmesan C., Beebee T.J.C., Fromentin J.-M., Hoegh-Guldberg O., Bairlein F. (2002). Ecological Responses to Recent Climate Change. Nature.

[34] Diamond S.E., Cayton H., Wepprich T., Jenkins C.N., Dunn R.R., Haddad N.M., Ries L. (2014). Unexpected Phenological Responses of Butterflies to the Interaction of Urbanization and Geographic Temperature. Ecology.

[35] Parmesan C. (2003). Chapter 24. Butterflies as Bioindicators for Climate Change Effects. Butterflies: Ecology and Evolution Taking Flight.

[36] Roy D.B., Sparks T.H. (2000). Phenology of British Butterflies and Climate Change. Glob. Change Biol..

[37] Betzholtz P.-E., Forsman A., Franzén M. (2023). Associations of 16-Year Population Dynamics in Range-Expanding Moths with Temperature and Years since Establishment. Insects.

[38] Hodgson J.A., Thomas C.D., Oliver T.H., Anderson B.J., Brereton T.M., Crone E.E. (2011). Predicting Insect Phenology across Space and Time: PREDICTING INSECT PHENOLOGY. Glob. Change Biol..

[39] Scriber J.M., Maher E., Aardema M.L. (2012). Differential Effects of Short Term Winter Thermal Stress on Diapausing Tiger Swallowtail Butterflies (*Papilio* spp.). Insect Sci..

[40] Singer M.C., Parmesan C. (2010). Phenological Asynchrony between Herbivorous Insects and Their Hosts: Signal of Climate Change or Pre-Existing Adaptive Strategy?. Philos. Trans. R. Soc. B Biol. Sci..

[41] Bale J.S., Hayward S.A.L. (2010). Insect Overwintering in a Changing Climate. J. Exp. Biol..

[42] Diamond S.E., Frame A.M., Martin R.A., Buckley L.B. (2011). Species’ Traits Predict Phenological Responses to Climate Change in Butterflies. Ecology.

[43] Wepprich T., Henry E., Haddad N.M. (2025). Voltinism Shifts in Response to Climate Warming Generally Benefit Populations of Multivoltine Butterflies. Ecol. Lett..

[44] Enriquez T., Visser B. (2023). The Importance of Fat Accumulation and Reserves for Insect Overwintering. Curr. Opin. Insect Sci..

[45] Stefanescu C., Herrando S., Páramo F. (2004). Butterfly Species Richness in the North-west Mediterranean Basin: The Role of Natural and Human-induced Factors. J. Biogeogr..

[46] Lafranchis T., Jutzeler D., Guillosson Y.J., Kan P., Kan B. (2015). La Vie des Papillons. Ecologie, Biologie et Comportement des Rhopalocéres de France.

[47] Gallou A., Baillet Y., Ficetola G.F., Després L. (2017). Elevational Gradient and Human Effects on Butterfly Species Richness in the French Alps. Ecol. Evol..

[48] Altermatt F. (2012). Temperature-related Shifts in Butterfly Phenology Depend on the Habitat. Glob. Change Biol..

[49] Kharouba H.M., Lewthwaite J.M.M., Guralnick R., Kerr J.T., Vellend M. (2019). Using Insect Natural History Collections to Study Global Change Impacts: Challenges and Opportunities. Philos. Trans. R. Soc. B Biol. Sci..

[50] Meineke E.K., Davies T.J., Daru B.H., Davis C.C. (2019). Biological Collections for Understanding Biodiversity in the Anthropocene. Philos. Trans. R. Soc. B Biol. Sci..

[51] Rákosy L. (2024). A Field Guide to the Butterflies of Romania.

[52] Devictor V., Van Swaay C., Brereton T., Brotons L., Chamberlain D., Heliölä J., Herrando S., Julliard R., Kuussaari M., Lindström Å. (2012). Differences in the Climatic Debts of Birds and Butterflies at a Continental Scale. Nat. Clim. Change.

[53] Popescu-Gorj A. (1964). Catalogue de La Collection de Lépidoptères “Prof. A. Ostrogovich” Du Muséum d’histoire Naturelle “Grigore Antipa” Bucarest.

[54] Rákosy L. (1988). A Valuable Collection of Lepidoptera in the Zoological Museum of the University in Cluj-Napoca. Stud. Univ. Babeş-Bolyai Biol..

[55] Rákosy L., Lászlóffy Z. (1997). Fauna de Macrolepidoptere de La Fânatele Clujului (Lepidoptera) (Cluj, Romania). Bul. Inf. Soc. Lepid. Rom..

[56] Askeyev O.V., Sparks T.H., Askeyev I.V., Tishin D.V., Tryjanowski P. (2010). East versus West: Contrasts in Phenological Patterns?. Glob. Ecol. Biogeogr..

[57] Lancaster L.T. (2016). Widespread Range Expansions Shape Latitudinal Variation in Insect Thermal Limits. Nat. Clim. Change.

[58] Menzel A., Yuan Y., Matiu M., Sparks T., Scheifinger H., Gehrig R., Estrella N. (2020). Climate Change Fingerprints in Recent European Plant Phenology. Glob. Change Biol..

[59] Toro-Delgado E., Vila R., Talavera G., Turner E.C., Hayes M.P., Horrocks N.P.C., Bladon A.J. (2024). Regional Differences in Thermoregulation between Two European Butterfly Communities. J. Anim. Ecol..

[60] Altermatt F. (2010). Climatic Warming Increases Voltinism in European Butterflies and Moths. Proc. R. Soc. B Biol. Sci..

[61] Donoso I., Stefanescu C., Martínez-Abraín A., Traveset A. (2016). Phenological Asynchrony in Plant–Butterfly Interactions Associated with Climate: A Community-wide Perspective. Oikos.

[62] Zografou K., Swartz M.T., Adamidis G.C., Tilden V.P., McKinney E.N., Sewall B.J. (2021). Species Traits Affect Phenological Responses to Climate Change in a Butterfly Community. Sci. Rep..

[63] Végvári Z., Juhász E., Tóth J.P., Barta Z., Boldogh S., Szabó S., Varga Z. (2015). Life-history Traits and Climatic Responsiveness in Noctuid Moths. Oikos.

[64] Wepprich T., Adrion J.R., Ries L., Wiedmann J., Haddad N.M. (2019). Butterfly Abundance Declines over 20 Years of Systematic Monitoring in Ohio, USA. PLoS ONE.

[65] Stocker T., Qin D., Plattner G., Tignor M., Allen S., Boschung J., Nauels A., Xia Y., Bex B., Midgley B. (2013). IPCC, 2013: Climate Change 2013: The Physical Science Basis. Contribution of Working Group I to the Fifth Assessment Report of the Intergovernmental Panel on Climate Change.

[66] Freimuth J., Bossdorf O., Scheepens J.F., Willems F.M. (2022). Climate Warming Changes Synchrony of Plants and Pollinators. Proc. R. Soc. B Biol. Sci..

[67] Tamura Y., Osawa T., Tabuchi K., Yamasaki K., Niiyama T., Sudo S., Ishigooka Y., Yoshioka A., Takada M.B. (2022). Estimating Plant–Insect Interactions under Climate Change with Limited Data. Sci. Rep..

[68] Kharouba H.M., Vellend M. (2015). Flowering Time of Butterfly Nectar Food Plants Is More Sensitive to Temperature than the Timing of Butterfly Adult Flight. J. Anim. Ecol..

[69] Baur J., Jagusch D., Michalak P., Koppik M., Berger D. (2022). The Mating System Affects the Temperature Sensitivity of Male and Female Fertility. Funct. Ecol..

[70] Sales K., Vasudeva R., Dickinson M.E., Godwin J.L., Lumley A.J., Michalczyk Ł., Hebberecht L., Thomas P., Franco A., Gage M.J.G. (2018). Experimental Heatwaves Compromise Sperm Function and Cause Transgenerational Damage in a Model Insect. Nat. Commun..

[71] Sales K., Vasudeva R., Gage M.J.G. (2021). Fertility and Mortality Impacts of Thermal Stress from Experimental Heatwaves on Different Life Stages and Their Recovery in a Model Insect. R. Soc. Open Sci..

[72] Thackeray S.J., Henrys P.A., Hemming D., Bell J.R., Botham M.S., Burthe S., Helaouet P., Johns D.G., Jones I.D., Leech D.I. (2016). Phenological Sensitivity to Climate across Taxa and Trophic Levels. Nature.

[73] Xiao H., Chen J., Chen L., Chen C., Wu S. (2017). Exposure to Mild Temperatures Decreases Overwintering Larval Survival and Post-Diapause Reproductive Potential in the Rice Stem Borer Chilo Suppressalis. J. Pest Sci..

[74] Löckinger M., Trutschnig W., Ulrich W., Gros P., Schmitt T., Habel J.C. (2024). Ecological Performance Determines Phenological Responses of Butterflies in Northern Austria. Glob. Ecol. Conserv..

[75] Zhang Z. (2023). Effects of Climate Change on Butterfly Species. Theor. Nat. Sci..

[76] Filazzola A., Matter S.F., Roland J. (2020). Inclusion of Trophic Interactions Increases the Vulnerability of an Alpine Butterfly Species to Climate Change. Glob. Change Biol..

[77] Gergelz P., Gór A., Hudák T., Ilonczai Z., Szombathely E. (2017). Nappali Lepkéink. Our Butterflies, in Hungarian.

[78] Höttinger H., Pendl M., Wiemers M., Pospisil A. (2013). Insekten in Wien ß Tagfalter.

[79] Kühn E., Musche M., Harpke A., Feldmann R., Ulbrich K., Wiemers M., Settele J. (2019). Tagfalter-Monitoring Deutschland: Jahresauswertung, 2018. Oedippus.

[80] Lee S., Jeon H., Kim M. (2020). Spatial Distribution of Butterflies in Accordance with Climate Change in the Korean Peninsula. Sustainability.

[81] Konvicka M., Kuras T., Liparova J., Slezak V., Horázná D., Klečka J., Kleckova I. (2021). Low Winter Precipitation, but Not Warm Autumns and Springs, Threatens Mountain Butterflies in Middle-High Mountains. PeerJ.

[82] Schulte T., Eller O., Niehuis M., Rennwald E. (2007). Die Tagfalter Der Pfalz, Bd. 1.—Flora Und Fauna in Rheinland-Pfalz. Beiheft.

[83] Ulrich W., Habel J.C., Gros P., Schmitt T. (2024). Recent Increasing Homogenisation in Austrian Butterfly Communities over the Past Decades. Oikos.

