# Supplementary material for: Long-Term Phenological Shifts in Butterfly Species from Transylvania, Romania—A Case Study
Source: Insects. 2025 Oct 20;16(10):1071. doi: 10.3390/insects16101071 (PMC12563528; doi:10.3390/insects16101071)
Supplement: Supplementary file 1 [file insects-16-01071-s001.zip › Figure S1.pdf]

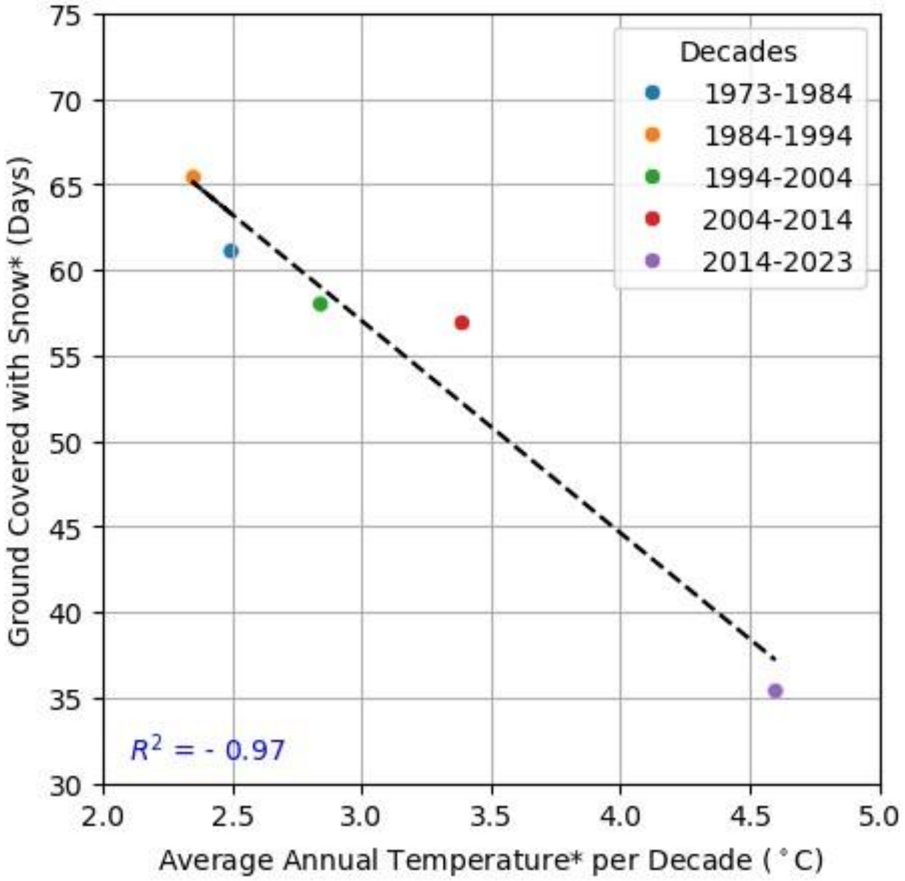

Figure S1. Relationship between the decline in snow cover and increasing average annual temperatures across decades (1973–2023). The interrupted line represents the linear fit through the data with a coefficient of determination R^2 proving a strong negative correlation.
